# Patterns of pleural pressure amplitude and respiratory rate changes during therapeutic thoracentesis

**DOI:** 10.1186/s12890-018-0595-7

**Published:** 2018-02-14

**Authors:** Monika Zielinska-Krawczyk, Elzbieta M. Grabczak, Marcin Michnikowski, Krzysztof Zielinski, Piotr Korczynski, Anna Stecka, Tomasz Golczewski, Rafal Krenke

**Affiliations:** 1 Department of Internal Medicine, Pulmonary Diseases & Allergy, Medical University of Warsaw, Banacha 1a, 02-097 Warsaw, Poland; 2 Nalęcz Institute of Biocybernetics and Biomedical Engineering, Polish Academy of Sciences, Ks. Trojdena 4, 02-109 Warsaw, Poland

**Keywords:** Pleural effusion, Pleural fluid, Pleural pressure, Thoracentesis, Pleural manometry, Pleural pathophysiology

## Abstract

**Background:**

Although the impact of therapeutic thoracentesis on lung function and blood gases has been evaluated in several studies, some physiological aspects of pleural fluid withdrawal remain unknown. The aim of the study was to assess the changes in pleural pressure amplitude (Ppl_ampl_) during the respiratory cycle and respiratory rate (RR) in patients undergoing pleural fluid withdrawal.

**Methods:**

The study included 23 patients with symptomatic pleural effusion. Baseline pleural pressure curves were registered with a digital electronic manometer. Then, the registrations were repeated after the withdrawal of consecutive portions of pleural fluid (200 ml up to 1000 ml and 100 ml above 1000 ml). In all patients the pleural pressure curves were analyzed in five points, at 0, 25%, 50%, 75% and 100% of the relative volume of pleural effusion withdrawn in particular patients.

**Results:**

There were 11 and 12 patients with right sided and left sided pleural effusion, respectively (14 M, 9F, median age 68, range 46–85 years). The most common cause of pleural effusion were malignancies (20 pts., 87%). The median total volume of withdrawn pleural fluid was 1800 (IQR 1500–2400) ml. After termination of pleural fluid withdrawal Ppl_ampl_ increased in 22/23 patients compared to baseline. The median Ppl_ampl_ increased from 3.4 (2.4–5.9) cmH_2_O to 10.7 (8.1–15.6) cmH_2_O (*p* < 0.0001). Three patterns of Ppl_ampl_ changes were identified. Although the patterns of RR changes were more diversified, a significant increase between RR at baseline and the last measurement point was found (*p* = 0.0097).

**Conclusions:**

In conclusion, therapeutic thoracentesis is associated with significant changes in Ppl_ampl_ during the respiratory cycle. In the vast majority of patients Ppl_ampl_ increased steadily during pleural fluid withdrawal. There was also an increase in RR. The significance of these changes should be elucidated in further studies.

**Trial registration:**

ClinicalTrial.gov, registration number: NCT02192138, registration date: July 1st, 2014.

## Background

Large volume pleural effusion leads to an increase in pleural pressure, negatively affects lung volumes and induces clinical symptoms (e.g. dyspnea and cough) [1–3]. Conversely, therapeutic thoracentesis usually results in a decrease in pleural pressure and has beneficial effects on pulmonary function [2, 4, 5]. Although the number of thoracenteses performed in the USA is reported between 127,000 and 173,000 procedures per year [6, 7], some physiological aspects of pleural fluid removal have not been adequately studied. This is partly because there is no universal and commonly accepted animal model for studying pleural pathophysiology. The relationship between pleural fluid volume and pulmonary function has been evaluated in several human studies. These studies showed that the increase in forced expiratory volume at first second (FEV_1_) and forced vital capacity (FVC) after therapeutic thoracentesis approximates 20–30% of the withdrawn pleural fluid volume [4, 8–10]. Changes in spirometric parameters in patients undergoing therapeutic thoracentesis were related to pleural pressure (Ppl). In a study by Light et al. [4], higher post-thoracentesis Ppl and smaller Ppl decrease after pleural fluid removal were associated with a more significant improvement in FVC.

Some other studies focused on the effect of therapeutic thoracentesis on blood gases. A study by Brandstetter and Cohen [11] showed that PaO_2_ decreases significantly early after therapeutic thoracentesis and returns to pre-thoracentesis value after 24 h. Different results were reported in later studies. Perpina et al. found a significant increase in PaO_2_ after pleural fluid removal reaching a maximum at 24 h, while Agusti et al. reported no significant effect of therapeutic thoracentesis on blood gases [12, 13]. Changes in blood gases after therapeutic thoracentesis were also studied in relation to pleural pressure and pleural elastance [14, 15]. Chen et al. reported a significantly larger increase in PaO_2_ and PaO_2_/FiO_2_ ratio in patients with normal pleural elastance (PE < 14 cmH_2_O/L) compared to patients with high (> 14 cmH_2_O/L) pleural elastance.

The use of digital pleural manometers not only allows to precisely assess pleural elastance during pleural fluid removal but also enables monitoring Ppl changes in different phases of the respiratory cycle. In our previous studies we observed some changes in Ppl curve characteristics during pleural fluid withdrawal [16, 17]. These included an increase in Ppl amplitude during the respiratory cycle and changes in respiratory rate. To our knowledge, this effect has not been evaluated and described in details so far. Therefore, we undertook a study aimed at the evaluation of changes in the respiratory pattern and pleural pressure amplitude associated with therapeutic thoracentesis.

## Methods

### Study design

This prospective, study was performed between January 2016 and August 2016. Consecutive patients with large volume pleural effusion referred to our department to perform therapeutic thoracentesis were enrolled. The study protocol was approved by the Institutional Review Board (KB 105/2012) and registered at ClinicalTrial.gov (NCT02192138). As the study was a part of larger project, the patients signed an informed consent for pleural pressure monitoring during and after therapeutic thoracentesis, as well as for all additional monitoring procedures (e.g. lung function) included in the study protocol.

### Patients

The specific inclusion criteria were as follows: (1) age between 18 and 85 years, (2) pleural effusion occupying at least one third of the ipsilateral hemithorax in posteroanterior chest radiograph (CXR), (3) no contraindications for therapeutic thoracentesis, (4) general health condition allowing prolonged procedure of therapeutic thoracentesis.

### Methods

Therapeutic thoracentesis was performed in sitting position. Small bore pleural catheter (outer diameter 8 Ch, i.e. 2.67 mm, length 12.5 cm; Turkel™ Safety System, Covidien, Whiteley Fareham, UK) was inserted to the pleural cavity in the dependent region under real-time ultrasound guidance. Pressure transducer and electronic manometer were connected to the pleural catheter via 3-way stopcock and carefully purged of air with sterile saline as described elsewhere [16]. The vertical reference point for a pressure of zero was defined at the level of catheter insertion into the chest. Then, the 3-way stopcock was connected to the pleural catheter and baseline pleural pressure curve was registered before beginning of pleural fluid withdrawal. Pleural fluid was aspirated with a 60 ml syringe. Pleural pressure curve was subsequently registered after withdrawal of each 200 ml of pleural fluid up to a total volume of 1000 ml. The duration of pleural pressure registration in each volume point was 60 s. When the volume of the removed fluid exceeded 1000 ml, registrations were performed after removal of each 100 ml. Vital signs and symptoms were registered together with pleural pressure changes. The pleural fluid withdrawal was terminated when one of the following occurred: (1) there was no more fluid in the pleural cavity, (2) poor procedure tolerance, i.e. onset or worsening of symptoms (e.g. severe dyspnea, chest pain, tachycardia, hemodynamic instability) with or without significant drop in Ppl (< − 20 cmH_2_0). The pleural catheter was fixed to the chest wall, purged with sterile saline and left in place for subsequent measurements not reported in this article.

**Fig. 1:**
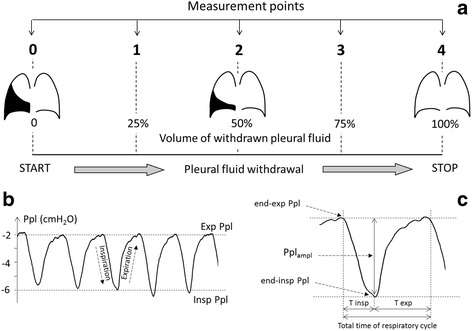
Methods of pleural pressure measurement and analysis. **a** Five points selected for analysis and their relation to the relative volume of removed pleural fluid (0, 25%, 50%, 75% and 100% of total removed volume). **b** An example of pleural pressure curve reflecting five respiratory cycles that was selected for analysis in each point. **c** Enlarged pleural pressure curve registered during one respiratory cycle and parameters that were measured: end-exp Ppl – end-expiratory pleural pressure, end-insp Ppl – end-inspiratory pleural pressure, T insp – inspiratory time, T exp. – expiratory time, Ppl_ampl_ – pleural pressure amplitude during a respiratory cycle

Pleural pressure curves were recorded during quiet tidal breathing. A special software was developed to perform a reliable analysis after the completion of pleural fluid withdrawal. In all patients the pleural pressure curves were analyzed in five points that reflected the relative volume of withdrawn pleural effusion, i.e. % of the total volume of pleural fluid removed in a particular patient (Fig. 1a). First, baseline data were assessed and then the calculations were repeated after 25%, 50%, 75% and 100% of total fluid volume withdrawn in a particular patient. In each measurement point at least five consecutive and comparable cycles of pleural pressure changes were selected for analysis (Fig. 1b). The following parameters were calculated: respiratory rate, mean and median pleural pressure (mean Ppl, median Ppl), end-expiratory pleural pressure (end-exp Ppl), end-inspiratory pleural pressure (end-insp Ppl), pleural pressure amplitude during a respiratory cycle (Ppl_ampl_ = end-exp Ppl – end-insp Ppl), total time of respiratory cycle (Fig. 1c). Also, pleural elastance was calculated as the change in pleural pressure (the difference between the baseline and closing pressure) divided by the volume of withdrawn pleural effusion [2, 3, 15].

### Statistical analysis

Data are presented as medians and interquartile ranges (IQRs, 25th to 75th percentiles). Statistical analysis was performed using Statistica 12.0 (StatSoft Inc., Tulsa, USA) and MedCalc Statistical Software version 13.2.2 (MedCalc Software bvba, Ostend, Belgium). Quantitative data distribution was assessed using the Shapiro-Wilk test. Analysis of variance of Ppl_ampl_ and RR in predefined points reflecting the relative volume of withdrawn pleural fluid was performed using Friedman’s test. The Wilcoxon test was used to compare the differences between parameters in two different points of assessment. The differences between continuous variables in two independent groups were tested using the non-parametric Mann–Whitney U-test. The strength and direction of the linear relationship between two variables was measured with Spearman’s rank correlation coefficient. All *P* values were 2-tailed and *P* < 0.05 was considered statistically significant.

## Results

Twenty-six patients were initially enrolled. However, three patients had to be excluded from final analysis due to low quality of measurements, i.e. high variability of pleural pressure curve that did not allow reliable calculation of Ppl and Ppl_ampl_. Thus, the investigated group included 23 patients (14 M, 9F, median age 68, range 46–85). There were 11 patients with right- and 12 patients with left-sided pleural fluid. Malignant, parapneumonic and rheumatoid pleural effusion was diagnosed in 20, 2 and 1 patient, respectively.

### The volume of pleural fluid removed in different points of assessment and pleural elastance

The data on the volume of pleural fluid removed in five different points of assessment are presented in Table 1. The median of total volume of pleural fluid withdrawn in 23 patients was 1800 (1500–2400) ml.

**Table 1:** The volume of removed pleural fluid in different points of assessment

Volume of removed PF	Point of assessment				
	0	1 (25%)	2 (50%)	3 (75%)	4 (100%)
median (ml)	0	450	900	1350	1800
IQR (25–75 percentile) (ml)	0	375–600	750–1200	1125–1800	1500–2400

The median elastance in the whole group was 8.56 (3.72–11.77) cmH_2_O/L. In 18 patients the elastance was lower than 14.5 cmH_2_O/L, in 3 it was slightly elevated (15.8, 16.9 and 17.8 cmH_2_O/L), while in 2 other it was very high (76.51 and 104.9 cmH_2_O). In these 2 last patients trapped lung was diagnosed. In 2 other patients small asymptomatic pneumothoraces were found in chest radiographs performed after the completion of the procedure. Hence, in these patients pleural elastance was low, pneumothorax *ex vacuo* was rather unlikely.

### Changes in pleural pressure amplitude (Ppl_ampl_) during pleural fluid withdrawal

**Fig. 2:**
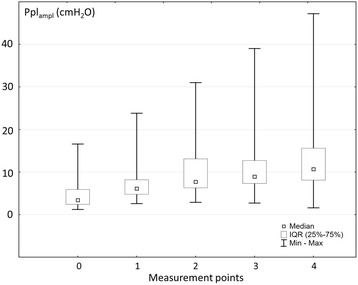
Changes in pleural pressure amplitude during pleural fluid (PF) withdrawal. Assessment points: 0 – baseline (before PF withdrawal), 1 - after aspiration of 25% of total PF volume, 2 - after aspiration of 50% of total PF volume, 3 - after aspiration of 75% of total PF volume, 4 - after aspiration of 100% of total PF volume

Significant unidirectional changes in Ppl_ampl_ were found during pleural fluid withdrawal (Friedman test, *p* < 0.0001) (Fig. 2). The median Ppl_ampl_ increased from 3.4 (2.4–5.9) cmH_2_O at baseline (point 0), to 10.7 (8.1–15.6) cmH_2_O after termination of thoracentesis (point 4).

A highly significant increase in Ppl_ampl_ was demonstrated between all pairs of measurements, except those between point 2 and 3 as well as 3 and 4 (Table 2). Even though the differences between Ppl_ampl_ in points 2 vs 3 and 3 vs 4 were not statistically significant, there was a numerical increase in Ppl_ampl_ between those points: 7.7 (6.3–13.1) vs 8.9 (7.3–12.7) cmH_2_O and 8.9 (7.3–12.7) vs 10.7 (8.1–15.6) cmH_2_O, respectively.

**Table 2:** Differences between Ppl_ampl_ in particular points of measurements

**Assessment point**	**0**	**1**	**2**	**3**	**4**
**0**	X	**p < 0.0001**	**p < 0.0001**	**p < 0.0001**	**p < 0.0001**
**1**	**p < 0.0001**	X	***p*** **< 0.0001**	***p*** **= 0.0002**	***p*** **= 0.0001**
**2**	**p < 0.0001**	**p < 0.0001**	X	*p* = 0.0945	***p*** **= 0.0047**
**3**	**p < 0.0001**	**p = 0.0002**	p = 0.0945	X	*p* = 0.0573
**4**	**p < 0.0001**	**p = 0.0001**	**p = 0.0047**	p = 0.0573	X

Each point of assessment (rows) was compared with all next points of assessment (columns). P for all pairs (Wilcoxon test) are presented with *P* values < 0.05 shown in bold

**Fig. 3:**
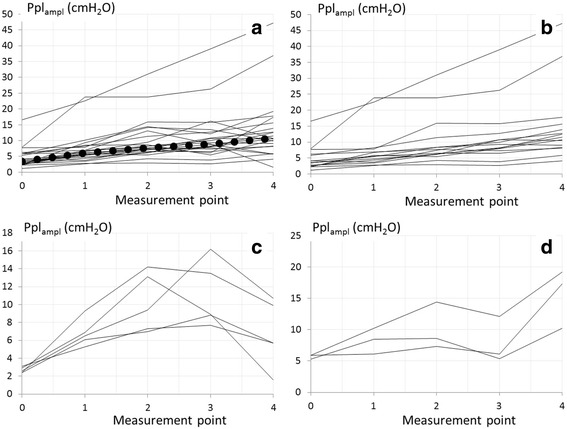
Curves representing changes in Ppl_ampl_ during pleural fluid withdrawal. **a** All patients (dotted line represents medians). **b** Patients with systematic increase of Ppl_ampl_ in all consecutive measurement points. **c** Patients with initial increase in Ppl_ampl_ followed by Ppl_ampl_ decline. **d** Patients with a general increase in Ppl_ampl_ interrupted by its transitional decrease

Curves representing changes in Ppl_ampl_ during therapeutic thoracentesis in individual patients are shown in Fig. 3a. Three slightly different patterns of Ppl_ampl_ changes were found. In 2/3 of patients (15/23), a systematic increase in all consecutive points of measurement was noted (Fig. 3b). In 5 patients there was an increase in Ppl_ampl_ between 0 and 2nd point or 0 and 3rd point with decrease in the last phase of the procedure (Fig. 3c). In 3 patients Ppl_ampl_ increased during the procedure with transitional drop between point 2 and 3 (Fig. 3d).

### Changes in RR during pleural fluid withdrawal

**Fig. 4:**
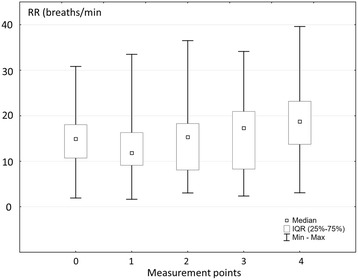
Changes in respiratory rate (RR) during pleural fluid (PF) withdrawal. PF – pleural fluid; assessment points: 0 – baseline (before PF withdrawal), 1 - after aspiration of 25% of total PF volume, 2 - after aspiration of 50% of total PF volume, 3 - after aspiration of 75% of total PF volume, 4 - after aspiration of 100% of total PF volume

Significant changes in RR were found during pleural fluid withdrawal (Friedman’s test, *p* < 0.0001) (Fig. 4). The median RR increased from 24.9 (20.7–28.0) per minute at baseline to 28.7 (23.7–33.7) per minute after termination of thoracentesis (point 4).

A significant increase between RR measured in points 0, 1, 2, 3 and that found in point 4 was demonstrated (Table 3). The transitional decrease in RR between points 0 and 1 (seen in Fig. 4) 24.9 (20.7–28.0) vs 21.8 (19.1–26.3) was statistically insignificant (Table 3, Wilcoxon test, *p* = 0.1070). There was a systematic and significant increase between RR measured in point 1 and all consecutive points.

**Table 3:** Differences between respiratory rate (RR) in particular points of measurements

**Assessment point**	**0**	**1**	**2**	**3**	**4**
**0**	X	p = 0.1070	*p* = 0.3458	*p* = 0.1485	***p*** **= 0.0097**
**1**	*p* = 0.1070	X	***p*** **= 0.0284**	***p*** **= 0.0039**	**p = 0.0001**
**2**	p = 0.3458	**p = 0.0284**	X	*p* = 0.3019	***p*** **= 0.0024**
**3**	p = 0.1485	**p = 0.0039**	p = 0.3019	X	***p*** **= 0.0192**
**4**	**p = 0.0097**	**p = 0.0001**	**p = 0.0024**	**p = 0.0192**	X

Each point (column 1) was compared with all the next points of assessment. P for all pairs (Wilcoxon test) are presented with *P* values < 0.05 shown in bold

**Fig. 5:**
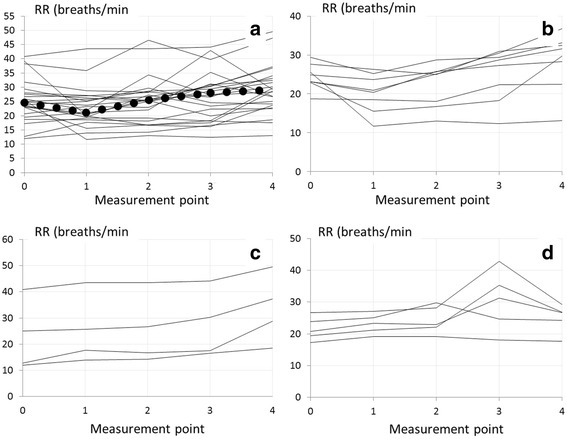
Curves representing changes in respiratory rate (RR) during pleural fluid withdrawal. **a** All patients (dotted line represents medians). **b** Patients with initial decline followed by increase of RR in the next measurement points. **c** Patients with small, but steady increase in RR. **d** Patients with stable RR followed by its increase in 2nd or 3rd point and decrease at the termination of the procedure

Figure 5 shows curves representing changes in RR during therapeutic thoracentesis in individual patients. In Fig. 5a all curves are presented, while Fig. 5b-d show curves with relatively similar characteristics.

There were no statistically significant correlations between Ppl_ampl_ and RR in any of the measurement points. However, highly significant correlations between pleural Ppl_ampl_ and pleural elastance were demonstrated with r and P reaching 0.67 and 0.0006, respectively, in the last point of assessment (point 4). Also, significant negative correlations between Ppl_ampl_ and mean Ppl in points 3 and 4 were found (*r* = − 0.596, *P* = 0.0034 and *r* = − 0.633, *P* = 0.0016; respectively). We did not find any significant and reliable differences between clinical parameters in patients with different patterns of Ppl_ampl_ changes. In particular, there was no difference between the pleural elastance (*p* = 0.3342), systolic and diastolic blood pressure, and respiratory rate.

## Discussion

Our study showed different patterns of Ppl_ampl_ and RR changes during pleural fluid withdrawal. Despite an extensive search of the literature, we could not find any earlier publications reporting the above aspects of therapeutic thoracentesis. The only study that reported Ppl_ampl_ measurements (described as pleural pressure swings) was that of Boshuizen et al. However, the authors presented only the difference between Ppl_ampl_ after removal of 200 ml of pleural fluid in terms of expandable vs unexpandable lung [18]. Hence, we believe, our paper may be the first report on Ppl_ampl_ during therapeutic thoracentesis.

The principle finding of the study is a relatively consequent increase in Ppl_ampl_ that follows pleural fluid withdrawal. The Ppl_ampl_ at the end of thoracentesis was higher in all but one patient (95.6%) compared to Ppl_ampl_ measured directly after pleural catheter insertion. In 65% of patients the Ppl_ampl_ increased steadily during the procedure. In 13%, there was a transient decrease in the third phase (between points 2 and 3) of the procedure with further significant increase in the fourth phase (between points 3 and 4), while in the remaining 22% patients steady increase in Ppl_ampl_ was noted in the first two or three phases with a decrease in the last phase of the procedure. Thus, we believe, three different patterns of Ppl_ampl_ changes during pleural fluid withdrawal can be distinguished. When patients with pattern 1 (steady increase) were compared to patients with pattern 3 (initial increase followed by a decrease) in terms of withdrawn pleural fluid volume, pleural pressure changes during the procedure and other parameters, no differences between these two groups were noted, except median Ppl_ampl_ at the completion of pleural fluid withdrawal (11.5 IQR 8.3–15.6 vs 5.7 IQR 5.7–9.9, respectively; *p* = 0.04). When patients were divided into two groups based on median baseline Ppl_ampl_ (below 3.4 and above 3.4 cm H_2_O) we found that the volume of withdrawn pleural fluid and pleural elastance were lower in low median baseline Ppl_ampl_ group (1760 vs 2150 ml and 8.05 vs 12.68 cmH_2_O/L, respectively). Hence, we may speculate about the potential relationship between these parameters and the difference between Ppl_ampl_ at the end of pleural fluid withdrawal.

The variability in RR changes during the procedure was noticeably higher than that found for Ppl_ampl_. In general, an increase in RR was found between the first (baseline) and the last measurement point (*p* = 0.0097). This was the case in 20/23 (87%) patients. The most common pattern of RR changes during pleural fluid withdrawal was its initial decrease followed by a steady increase. Nonetheless, this pattern was demonstrated in only approximately 1/3 of patients. The other characteristics of RR changes found in 17% and 22% of patients were steady and slow increase from point 0 to point 1 and initial increase with decrease in the last point (or two last points) of measurements, respectively. In the remaining 26% of patients different, difficult to categorize RR changes were observed.

As there were no earlier studies on Ppl_ampl_ changes during therapeutic thoracentesis, we could not compare our results with other reports. The data on RR changes are also scarce with one study showing a decrease in RR from 19.4± 6.5 to 15.5± 6.3 after drainage of significant pleural effusion [19]. It should be emphasized, however, that the above study was performed in critically ill surgical patients who were on volume-regulated mechanical ventilation.

The paucity of data, together with the initial character of our report and relatively small study group, makes the explanation of our findings challenging. It could have been hypothesized that the overall increase in the respiratory system compliance associated with pleural fluid removal would result in decreased pleural pressure amplitude rather than in its increase. With higher respiratory system compliance, lower transpulmonary pressure should be sufficient to maintain or even increase tidal volume and minute ventilation. On the other hand, it could have been argued that the changes in Ppl_ampl_ would supposedly be mainly associated with the elastic properties of the lung and visceral pleura. If so, the expansion of lung elastic elements associated with increasing lung volume due to decreasing Ppl that follows pleural fluid withdrawal would result in higher transpulmonary pressure required to maintain tidal volume and minute ventilation. The decline in lung compliance resulting from stretching of its elastic elements associated with lung re-expansion may be particularly significant in the atelectatic lung characterized by relative surfactant deficiency or dysfunction. We believe, the above reasoning is fully consistent with our findings of increasing Ppl_ampl_ and RR during therapeutic thoracentesis. Significant negative correlation between Ppl_ampl_ and Ppl and positive correlation between Ppl_ampl_ and pleural elastance may further support the correctness of our interpretations.

We think that even if the differences in RR were relatively small and their clinical relevance might be questioned, our findings may raise new questions on dyspnea perception, changes in voluntary ventilation and work of breathing after therapeutic thoracentesis. Although pleural fluid withdrawal reduces dyspnea in nearly all patients, the mechanism of this phenomenon is unclear. As improvement in lung function parameters is relatively small and data on blood gases are equivocal, it has been postulated that this is mainly related to the reduced pressure on the diaphragm and subsequent restoration of its normal shape which enables inspiratory muscles to work on a more advantageous position [20, 21]. One study showed that during pleural fluid drainage, the neural impulses from mechanoreceptors located in muscles, chest wall and lung parenchyma significantly contributed to the perception of breathlessness and indirectly influenced the respiratory rate [22]. Paradoxically, the increasing Ppl_ampl_ during therapeutic thoracentesis found in our study may suggest an increased work of breathing rather than its reduction. It is not known whether the increased Ppl_ampl_ is associated with increased tidal volume and minute ventilation. These, and other questions should be answered in further studies which would involve continued measurement of air flow through the airways enabling estimation of tidal volume and the minute ventilation.

A prerequisite for our study on the physiological effects of pleural fluid and therapeutic thoracentesis is pleural manometry. Although pleural pressure measurement was commonly applied to create and maintain artificial pneumothorax used to treat tuberculosis a century ago, its modern use in studying pleural pathophysiology began in early 1980s and was associated with impressive results presented by Light and colleagues [23]. The authors demonstrated three different patterns of pleural elastance which were further confirmed in other studies [24–26]. However, water manometers or modified overdamped water manometers used in earlier studies had some significant limitations. They did not allow either for the observation of pleural pressure oscillations associated with respiratory cycle or for the precise measurement and registration of instantaneous pleural pressure [25]. Introduction of new systems based on electronic pressure transducers opened new possibilities to study pleural pathophysiology during pleural fluid removal [14, 26–29]. This refers not only to the measurement of instantaneous pleural pressure but also to data display, registration and analysis [2, 16, 18, 30]. Electronic manometers had very low inertia and high frequency of measurements (50 Hz) allowing reliable measurements of pleural pressure even during cough [17]. The use of electronic systems of pleural pressure registration allows very precise and detailed analysis of pleural pressure curve, as showed in our study and the paper by Boshuizen et al. [18]. We believe that further developments in monitoring of different parameters of respiratory system will help to precisely explain the pathophysiological effects of pleural fluid accumulation and its removal.

We are aware about some limitations of our study. First, the investigated group was relatively small. The limited number of patients does not allow a full assessment of Ppl_ampl_ and RR characteristics in subgroups presenting different patterns of changes. Very small groups of patients with inconsecutive increase in Ppl_ampl_ (only 3 patients) and Ppl_ampl_ decrease in the last phase of the procedure (5 patients) do not allow a reliable comparative analysis between of different variables in those groups. Second, as the vast majority of our patients had malignant pleural effusion we cannot be sure that the characteristics of Ppl_ampl_ and RR changes would be similar in patients with non-malignant pleural effusion. Third, the analysis of Ppl_ampl_ and RR changes in relation to the relative volume of withdrawn pleural effusion (% of the total volume removed in individual patients) may be questioned. However, this method of analysis was applied to make our results relatively independent of the absolute volume of removed pleural fluid. This approach was based on the assumption that in all patients therapeutic thoracentesis is a complete procedure independently of the volume of the withdrawn pleural fluid and justified the comparison of the procedures of removal of different volumes of pleural fluid. In our opinion, the analysis of Ppl_ampl_ and RR as the function of absolute volume of removed pleural fluid in patients with removed pleural fluid volume ranging from 500 to 4250 ml would be even more controversial.

## Conclusions

In conclusion, therapeutic thoracentesis is associated with significant changes not only in pleural pressure but also in pleural pressure amplitude during the respiratory cycle. Contrary to pleural pressure, the pleural pressure amplitude steadily increases in the vast majority of patients. The significance of Ppl_ampl_ and RR changes associated with pleural fluid withdrawal should be elucidated in further studies.

## Abbreviations

CXR: chest radiograph
end-exp Ppl: end-expiratory pleural pressure
end-insp Ppl: end-inspiratory pleural pressure
FEV_1_: forced expiratory volume at first second
FVC: forced vital capacity
IQR: interquartile range
PE: pleural elastance
PF: pleural fluid
Ppl: pleural pressure
Ppl_ampl_: pleural pressure amplitude
RR: respiratory rate
